# Potential probiotic-associated traits revealed from completed high quality genome sequence of *Lactobacillus fermentum* 3872

**DOI:** 10.1186/s40793-017-0228-4

**Published:** 2017-02-01

**Authors:** Burhan Lehri, Alan M. Seddon, Andrey V. Karlyshev

**Affiliations:** School of Life Sciences, Pharmacy and Chemistry, SEC Faculty, Kingston University, Penryn Road, Kingston upon Thames, KT1 2EE UK

**Keywords:** Probiotics, *Lactobacillus fermentum*, Genome sequencing, Bacteriocin, Collagen binding protein, Mucus binding protein, Prophage

## Abstract

The article provides an overview of the genomic features of *Lactobacillus fermentum* strain 3872. The genomic sequence reported here is one of three *L. fermentum* genome sequences completed to date. Comparative genomic analysis allowed the identification of genes that may be contributing to enhanced probiotic properties of this strain. In particular, the genes encoding putative mucus binding proteins, collagen-binding proteins, class III bacteriocin, as well as exopolysaccharide and prophage-related genes were identified. Genes related to bacterial aggregation and survival under harsh conditions in the gastrointestinal tract, along with the genes required for vitamin production were also found.

**Electronic supplementary material:**

The online version of this article (doi:10.1186/s40793-017-0228-4) contains supplementary material, which is available to authorized users.

## Introduction

Probiotics are widely used for treatment of auto-immune conditions including allergic reactions, as well as metabolic disorders and are being applied as alternatives or additives to antibiotic treatment [1–3]. Probiotics may provide a beneficial effect by modulating the host immune system, via the release of antimicrobial substances, or through competitive exclusion of pathogenic bacteria [4]. Various bacteria belonging to the *Lactobacillus* genus (including *L. fermentum*) are commonly used as probiotics [5]. The efficacy of these bacteria is not only species-specific, but also varies between the strains of the same species. *Lactobacillus* bacteria have a generally accepted as safe status. They are commonly found in various food products and are a part of the normal flora in animals and humans [6]. However, some lactobacilli have been found to lower the intestinal barrier in vitro [7]. *L. fermentum* 3872 has been patented in Russia along with a consortium of other Lactobacilli relating to their antimicrobial and probiotic uses [8]. *L. fermentum* 3872 was sequenced in order to determine molecular modes of actions that may potentially be used against pathogenic bacteria that live in the same habitat as strain 3872, along with genes relating to its ability to survive harsh conditions of the GIT. Genomic data relating to the microflora of humans are also important for better understanding the role these bacteria play within its natural environment. With more high quality genomic data being made available a consortium of probiotics with similar modes of action may be utilised to effectively combat pathogenic bacteria. Currently, the genome sequence of *L. fermentum* strain 3872 reported here is one of only three complete genome sequences deposited in GenBank, with genome sequences of 16 more strains either being incomplete (draft) or containing ambiguities. For example, the genome of strain CECT 5716 (GenBank accession number CP002033) is shown in the GenBank as ‘complete’ and circular despite having a large number of ambiguities in the sequence. The aim of this study was to determine and characterise a complete genome sequence of this microorganism and to identify its specific genetic features.

## Organism information

### Classification and features

**Fig. 1:**
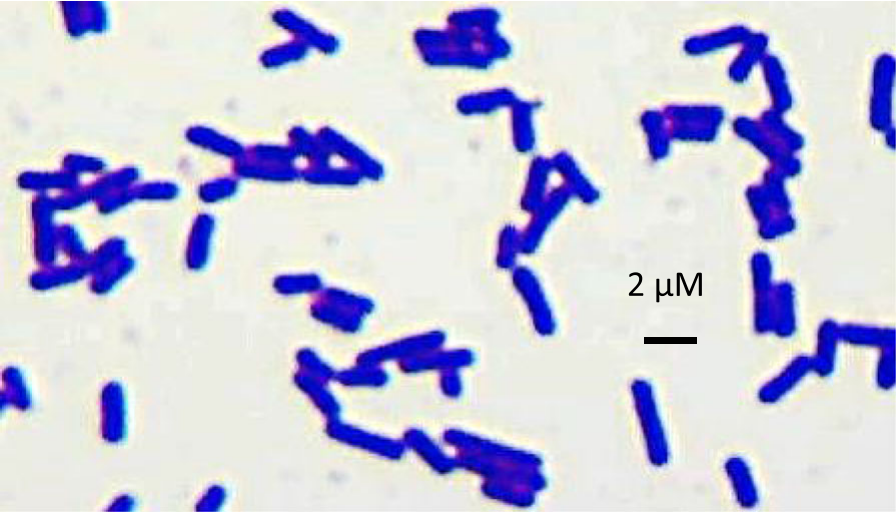
Photomicrograph of *L. fermentum* 3872 the bacteria was grown overnight at 37 °C using MRS agar and gram stained. The image was taken using an optical microscope with magnification 100 ×

**Fig. 2:**
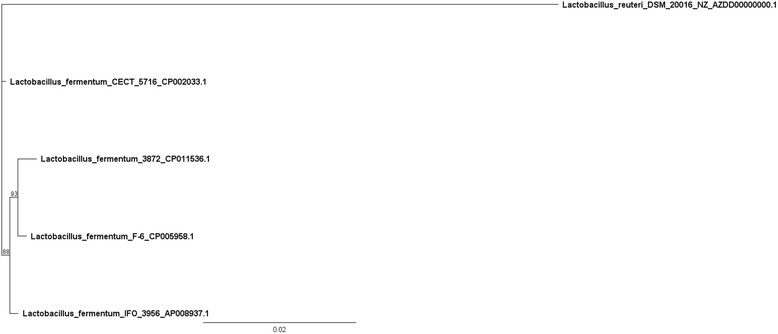
Phylogenetic tree based on comparative analysis of 16S rRNA genes. The sequences were aligned using the MUSCLE alignment tool [47]. The numbers above the tree nodes represent Bayesian posterior percentage probabilities computed using MrBayes 3.2.2 [48]. The tool used the HKY85 substitution model. A Markov Chain Monte Carlo chain length of 1,100,000 of a burn in length of 100,000, heated chains of 4 and a heated chain temperature of 0.2. *Lactobacillus_reuteri_*DSM_20016_NZ_AZDD00000000.1 was used as an out-group. The tree generated was further modified using Geneious tree builder [15]

*Lactobacillus fermentum* 3872 is a Gram-positive, rod-shaped (Fig. 1), facultative anaerobic bacteria [9] (Table 1). The strain is deposited under accession number VKM B-2793D at the All-Russian Collection of Microorganisms, Pushchino, Moscow Regions, Russia. Isolated from milk of a healthy woman. Identified as *Lactobacillus fermentum* in 2011 at the Institute of Engineering Immunology, Lyubuchany, Chekhov District, Moscow Regions, Russia. When grown in MRS agar *L. fermentum* 3872 forms medium sized, white colonies, that are round, smooth, and convex [8]. *L. fermentum* 3872 was isolated from the milk of a healthy human female and has been found in infant and mother fecal matter along with vaginal secretions, indicating the strains ability to be present in different human ecological habitats [8]. The bacterium has shown to be resistant to gastric and intestinal stresses, have high adhesion to human HeLa and buccal cells and has the ability to produce hydrogen peroxide and lactic acid, the release of which can be damaging to pathogenic bacteria [8]. *L. fermentum* 3872 when present with a mixture of probiotics has been found to be a promising tool for the treatment of mastitis [8]. *L. fermentum* 3872 belongs to the phylum firmicutes, among the circular genome sequences of *L. fermentum* the genome of strain 3872 appears to be most closely related to *L. fermentum* F6 (Fig. 2).

**Table 1:** Classification and general features of *Lactobacillus fermentum* 3872^T^ [38]

MIGS ID	Property	Term	Evidence code^a^
	Classification	Domain Bacteria	TAS [39]
		Phylum *Firmicutes*	TAS [40]
		Class *Bacilli*	TAS [41, 42]
		Order *Lactobacillales*	TAS [41, 42]
		Family *Lactobacillaceae*	TAS [43, 44]
		Genus *Lactobacillus*	TAS [44, 45]
		Species *Lactobacillus fermentum*	TAS [44, 45]
		(Type) strain: 3872^*T*^	
	Gram stain	Positive	IDA
	Cell shape	Rod	IDA
	Motility	Not known	
	Sporulation	Not known	
	Temperature range	30-42 °C	TAS [8]
	Optimum temperature	37 ± 2 °C	TAS [8]
	pH range; Optimum	not known; 5.5-6.0	TAS [8]
	Carbon source	D-Ribose, D-Galactose, D-Glucose, D-Fructose, D-Maltose, D-Lactose, D-Melibiose, D-Sucrose, D-Trehalose, D-Raffinose	TAS [8]
MIGS-6	Habitat	*Homo sapiens*; milk	TAS [8]
MIGS-6.3	Salinity	Not known	
MIGS-22	Oxygen requirement	Facultative anaerobe	TAS [8]
MIGS-15	Biotic relationship	commensal	TAS [8]
MIGS-14	Pathogenicity	None known	NAS
MIGS-4	Geographic location	Russia/Moscow region	TAS [8]
MIGS-5	Sample collection	2011	TAS [8]
MIGS-4.1	Latitude	Not known	
MIGS-4.2	Longitude	Not known	
MIGS-4.4	Altitude	Not known	

^a^Evidence codes - *IDA* inferred from direct assay, *TAS* traceable author statement (i.e., a direct report exists in the literature), *NAS* non-traceable author statement (i.e., not directly observed for the living, isolated sample, but based on a generally accepted property for the species, or anecdotal evidence). These evidence codes are from the Gene Ontology project [46]

## Genome sequencing information

### Genome project history

Determination of a draft genome sequence of *L. fermentum* 3872 allowed the identification of a number of genes that may potentially be involved in probiotic activity, including a gene encoding a collagen-binding protein [9]. The latter was subsequently found to be located on plasmid pLF3872, the sequence of which was reported in 2015 [10]. In addition to the *cbp* gene, this plasmid, also contained a number of conjugation-related genes, as well as two toxin-antitoxin gene pairs required for stable maintenance of the plasmid within the bacterial cell [10]. The current article conducts a detailed analysis of the recently completed chromosomal sequence of *L. fermentum* 3872, the assembly is of high quality due to the use of a hybrid sequencing approach along with a physical map of the genome described below. The article also conducts comparative analysis with other completed genome sequences belonging to the same species in order to determine targets for future probiotic experiments.

### Growth conditions and genomic DNA preparation

*L. fermentum* 3872 was grown at 37 °C overnight on MRS agar plates under anaerobic conditions. DNA was isolated using Gentra Puregene Yeast/Bact Kit (Qiagen). For IonTorrent sequencing the NanoView photometer result indicated DNA concentration of 347ug/ul with DNA quality of A260/A280: 1.922 and A260/A230: 1.881. For Pacbio sequencing the NanoView photometer result for the extracted DNA was 314 ng/ul, A260/A280: 1.78 and A260/A230: 1.43, the Qubit DNA concentration result was 318 ng/ul. The DNA quality was also assessed by using agarose gel electrophoresis which indicated high concentration and good quality DNA (data not shown).

### Genome sequencing and assembly

The complete circular genome sequence of *L. fermentum* 3872 was determined by employing a hybrid sequencing approach, including PacBio and IonTorrent PGM sequencing, as well as OpGen optical mapping. Long but high error and low coverage reads generated by PacBio were used as a scaffolding tool. PacBio sequencing was conducted using an RSII sequencing machine with P6/C4 sequencing chemistry and a single SMRT cell. HGAP and CELERA bioinformatics tools were used for the removal of low quality reads and generation of one large contig representing a circular 2.3 Mb chromosomal sequence of *L. fermentum* 3872. Short, but low error and high coverage reads produced by IonTorrent PGM using 314v2 chip and 400 bp kit were used for sequence verification and correction, which was essential for the low coverage areas. Three runs of IonTorrent sequencing were conducted producing 1,290,864 reads. Genome coverage by PacBio was 19.6 fold, as estimated by mapping of 4,902 reads between 500 and 21,671 bases long, with 4,871 of reads (99.37%) representing 99.07% nucleotides mapped. When combined with IonTorrent data, read mapping resulted in 413,661,861 bases (99.57%) mapped onto the assembly (2,330,492 nt) corresponding to 177.5 fold coverage (173.9 and 293.8 for chromosome and plasmid respectively). An optical map generated by OpGen technology was used for validation of the assembly, as well as for trimming and circularisation of the genome sequence. The genome information is summarised in Table 2 and Additional file 1: Table S1.

**Table 2:** Project information

MIGS ID	Property	Term
MIGS 31	Finishing quality	Completed high quality
MIGS-28	Libraries used	IonTorrent OT2 400 sequencing kit, PacBio P6/C4
MIGS 29	Sequencing platforms	Ion Torrent Personal Genome Machine, PacBio RSII sequencing Machine
MIGS 31.2	Fold coverage	19.7 (PacBio), 49.6 (Ion Torrent run1), 60.1 (Ion Torrent run 2), 47.9 (Ion Torrent run 3)
MIGS 30	Assemblers	CELERA, MIRA
MIGS 32	Gene calling method	NCBI PGAP, PROKKA, RAST, BASys
	Locus Tag	NZ_CP011536
	Genbank ID	CP011536.1
	GenBank Date of Release	28/5/2015
	GOLD ID	Ga0099330
	BIOPROJECT	PRJNA224116, PRJNA213970
MIGS 13	Source Material Identifier	VKM:B-2793D
	Project relevance	biotechnological, antimicrobial, probiotic

### Genome annotation

The genome sequence was annotated using PROKKA [11], BASys [12] and RAST [13] tools. In addition, the genome was annotated by NCBI GenBank annotation pipeline [14]. Some annotation irregularities (such as e.g. truncated coding sequences) produced by these four annotation tools were identified and corrected using Geneious software [15].

## Genome properties

The size of the *L. fermentum* 3872 genome (including the plasmid) is 2,330,492 bp. The G + C content of the circular chromosome (2,297,851 bp) is 55.6%. It contains 2328 genes, 2127 of which encode proteins and 128 are pseudogenes. There are 15 genes encoding rRNAs (23S, 16S and 5S) and 58 genes encoding tRNAs. The genome summary is presented in Tables 3, 4, 5.

**Table 3:** Summary of the genome: one chromosome and one plasmid

Label	Size (Mb)	Topology	INSDC identifier	RefSeq ID
Chromosome 1	2297851 bp	Circular	CP011536.1	NZ_CP011536.1
Plasmid 1	32641 bp	Circular	CP011537.1	NZ_CP011537.1

**Table 4:** Genome statistics

Attribute	Value	Percent
Genome size (bp)	2,330,492	100.00
DNA coding (bp)	2,028,095	87.02
DNA G + C (bp)	1,179,376	50.56
DNA scaffolds	2	100.00
Total genes	2,328	100.00
Protein coding genes	2127	91.37
RNA genes	73	3.14
Pseudo genes	128	0.05
Genes in internal clusters	481	20.66
Genes with function prediction	1824	78.35
Genes assigned to COGs	1563	67.14
Genes with Pfam domains	1898	81.53
Genes with signal peptides	37	1.59
Genes with transmembrane helices	507	21.78
CRISPR repeats	3	0.13

**Table 5:** Number of genes associated with general COG functional categories

Code	Value	Percent^a^	Description
J	179	8.42	Translation, ribosomal structure and biogenesis
A	0	0.00	RNA processing and modification
K	108	5.08	Transcription
L	97	4.56	Replication, recombination and repair
B	0	0.00	Chromatin structure and dynamics
D	26	1.22	Cell cycle control, Cell division, chromosome partitioning
V	34	1.60	Defense mechanisms
T	58	2.72	Signal transduction mechanisms
M	83	3.90	Cell wall/membrane biogenesis
N	11	0.52	Cell motility
U	13	0.61	Intracellular trafficking and secretion
O	52	0.02	Posttranslational modification, protein turnover, chaperones
C	72	3.39	Energy production and conversion
G	91	4.28	Carbohydrate transport and metabolism
E	148	6.96	Amino acid transport and metabolism
F	96	4.51	Nucleotide transport and metabolism
H	95	4.47	Coenzyme transport and metabolism
I	63	2.96	Lipid transport and metabolism
P	81	3.80	Inorganic ion transport and metabolism
Q	21	0.99	Secondary metabolites biosynthesis, transport and catabolism
R	134	6.30	General function prediction only
S	6.30	3.76	Function unknown
-	766	36.01	Not in COGs

^a^Based on the total number of protein encoding genes

## Insights from the genome sequence

**Fig. 3:**
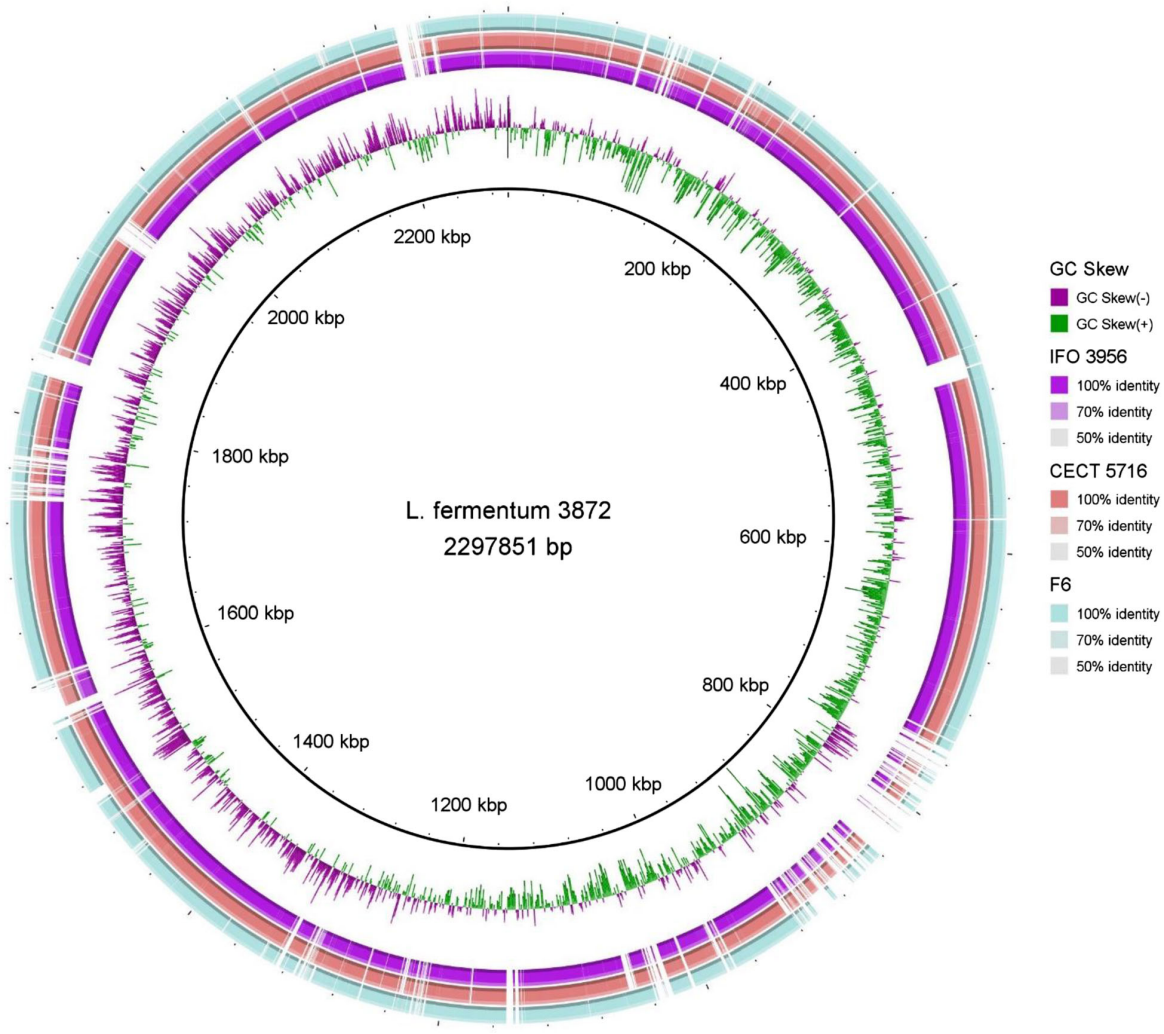
*L. fermentum* 3872 genome representation showing GC skew. Leading and lagging strands are shown in green and purple. BlastN comparison of the genome of *L. fermentum* 3872 against those IFO 3956, CECT 5716, and F6, are indicated by the colour coded key. The intensity of each colour indicates nucleotide percentage identity. The diagram was generated using BRIGS software [16] using an upper identity threshold of 70% and a lower identity threshold of 50%

The circular view of the chromosome of *L. fermentum* 3872 was generated by using BRIGS software [16]. The diagram indicates the leading (high G and low C region) and lagging (low G and high C region) strands of the *L. fermentum* 3872 chromosomal sequence (Fig. 3). Local GC skew deviations within the leading or lagging strand may indicate newly incorporated DNA, inversion or translocations [17]. The diagram shows comparison of the genomic sequence of *L. fermentum* 3872 with those of *L. fermentum* CECT 5716, IFO 3956 and F6 strains.

*L. fermentum* 3872 contains genes required for the synthesis of such vitamins as B1, B2, B5, B7 and B9. These genes may play a crucial role in providing the natural hosts with essential vitamins. There are symporter encoding genes that allow the bacteria to survive acidic conditions of the stomach and thrive within the gastrointestinal tract. Among such genes are those encoding Na^+^/H^+^ (four copies), as well as gluconate/H^+^, sugar/H^+^, amino acid/H^+^, and glutamate/H^+^ symporters.

Survival of lactic acid bacteria within the gut is dependent on sugar metabolism and amino acid decarboxylation/deamination assisting in maintaining optimal pH levels [18]. Among relevant genes of *L. fermentum* 3872 are those involved in arginine and proline metabolism (27 genes). There are also 14 genes involved in glutathione metabolism, which in *Lactobacillus salivarius* was found to be required for acid stress response [19]. There is a gene encoding dTDP-glucose 4,6-dehydratase (Locus tag: N573_RS00605). In *Lactobacillus plantarum* this protein was found to be associated with gastric acid tolerance [20]. There is a gene encoding Undecaprenyl-diphosphatase (EC 3.6.1.27) (Locus tag: N573_RS09665) with a possible role in bacitracin resistance by similarity to *E. coli* producing a similar protein [21]. In other bacteria, such as *Lactobacillus rhamnosus* [22], the genes encoding DnaK (*L. ferementum* 3872 Locus tag: N573_RS04975) and GroEL (*L. ferementum* 3872 Locus tag: N573_RS01895) are known to play a role in heat and hyperosmotic shock tolerance. In addition, in *Lactobacillus plantarum* both genes are also implicated in mucin binding [23] potentially inhibiting adherence of pathogenic bacteria to the mucus layer. Furthermore, the GroEL of *Lactobacillus johnsonii* La1 was found to be a cell surface located protein capable of inducing aggregation of a gastric pathogen *Helicobacter pylori* in vitro [24]. A gene (Locus tag: N573_RS03470) encoding a protein similar to *Lactobacillus johnsonii* La1 Translational Elongation Factor involved in bacterial adhesion to host cells [25], was also found. By similarity to function of similar genes found in *Lactobacillus plantarum* [23], *L. fermentum* 3872 genes encoding D-Lactate dehydrogenase (Locus tag: N573_RS11010) and 6-phosphogluconate dehydrogenase (Locus tag: N573_RS10960) are likely to promote bacterial adhesion to mucin and intestinal epithelial cells. There is a number of genes (e.g. loci N573_RS00495, N573_RS00500 and N573_RS00505, located to the same gene cluster) potentially involved in the biosynthesis of exopolysaccharides, which in other lactic acid bacteria were found to be important for bacterial survival and protection from toxic compounds [18].

### Comparative genomics

**Fig. 4:**
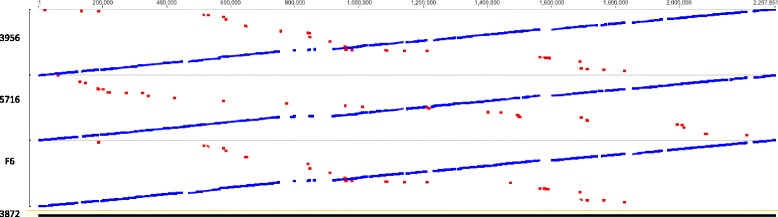
Comparison of the genomes of *L. fermentum* strains 3872, F6, 5716 and IFO 3956 using LASTZ program with a step length of 20 and a seed pattern of 12 of 19 [26]. Similar direct and inverted regions are shown in blue and red respectively

**Fig. 5:**
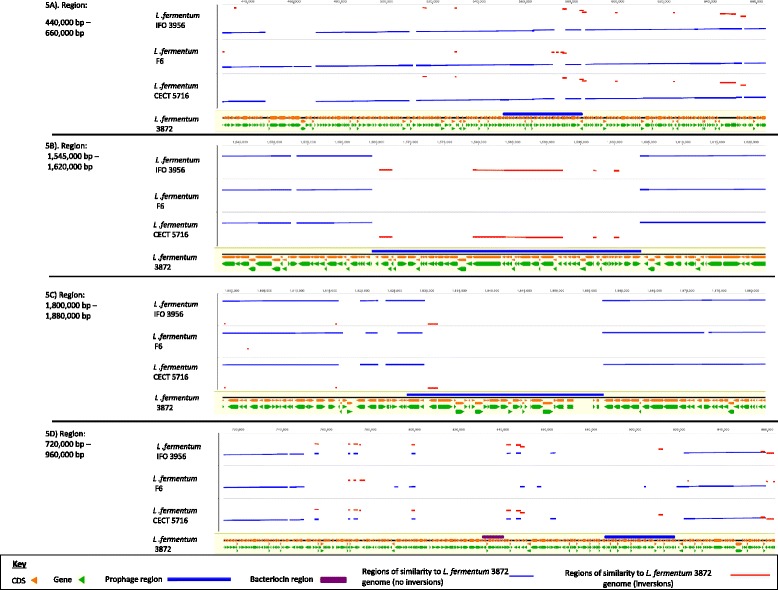
Comparison of the genomes of *L. fermentum* strains 3872, F6, CECT 5716 and IFO 3956 using LASTZ program with a step length of 20 and a seed pattern of 12 of 19 [26] with close-up of regions containing bacteriocin and prophages

Comparison of the complete chromosomal sequences of *L. fermentum* using LASTZ software [26] revealed a unique region of the *L. fermentum* 3872 genome (between positions 748,875 bp and 919,330 bp) (Fig. 4) This region contains genes encoding hypothetical proteins, enterolysin A (835,633 bp-836,847 bp) and ‘CAAX amino terminal protease self-immunity’ (838,683 bp-839,366 bp) protein, suggesting the bacterial ability to produce a bacteriocin. This was confirmed by running BAGEL3 bacteriocin prediction software [27], which identified a region (830,634 bp-840,633 bp) responsible for the biosynthesis of class III bacteriocin (Fig. 5d). No similarities were found for this region when using NCBI BlastN and the non-redundant database.

The region between 1,564,375 bp and 1,603,857 bp of the *L. fermentum* 3872 genome sequence contains inversions of respective parts of the genomes of *L. fermentum* strains F6, CECT 5716 and IFO 3956. This region also contains some prophage-related genes not found in the genomes of strains used for comparison. The region between 1,829,274 bp and 1,857,186 bp has a counterpart in *L. gasseri* ATCC 33323 (GenBank accession: CP000413) genome and may have been acquired via horizontal gene transfer (data not shown). The region between 2,212,692 bp and 2,237,160 bp has no matching sequences in the genomes of *L. fermentum* strains F6, CECT 5716 and IFO 3956, and contains conjugation and peptidoglycan hydrolase genes. NCBI BlastN analysis using the non-redundant database revealed high similarities to plasmid sequences, particularly with plasmid pPECL-5 from *Pediococcus claussenii* ATCC BAA-344 (e-value 0.0, query cover 55%). The other parts of this region contain the genes encoding transposases and an internalin J-like protein (InlJ, locus tag: N573_011130), containing an MucBP (mucin binding protein) domain [28, 29].

The genome of *L. fermentum* 3872 contains putative mucus binding protein-encoding gene also present in the genomes of strains F6, IFO 3956 and CECT 5716, but not in any other *Lactobacillus* genomes sequenced to date. Moreover, a gene, encoding a partial collagen-binding protein (Locus tag: N573_000435) is also found. This protein contains an LPXTG_anchor domain and a single B domain, but lacks the collagen-binding A domain [30]. The gene encoding this protein was not found in any other *L. fermentum* strain.

The *L. fermentum* 3872 genome also contains an aggregation substance precursor protein encoding gene (Locus tag: N573_004020). The gene may potentially contribute to bacterial adhesion and aggregation [31]. There are a number of exopolysaccharide production-related genes. In particular, *epsH* (Locus tag: N573_008790) predicted to be involved in biofilm formation, and may also contribute to protection against colitis [32]. Remarkably, neither of these two genes (Locus tags: N573_004020, N573_008790) are present in the genomes of the strains used for comparison. An enolase encoding gene (Locus tag: N573_002185) present in *L. fermentum* 3872 may promote bacterial adhesion to collagen [33].

Comparative analysis of the genomes of *L. fermentum* strains 3872, F6 and IFO 3956 using Spine/AGent Pan-Core genome analysis tools with default parameters [34] allowed the identification of 428 unique ORFs of the *L. fermentum* 3872 genome with further 1650 ORFs representing core genes. One hundred and forty eight of the unique ORFs encode hypothetical proteins, with the other genes representing mobile elements, CRISPR-related and those involved in conjugal transfer. Among other genes were those encoding ABC transporters and those involved in bacteriocin biosynthesis, heavy metal resistance, and prophage-related genes.

### Prophages

PHAST software [35] allowed the identification of four prophage related regions (Fig. 5), each containing a phage attachment (ATT) site. A 34.5 kb region between 550,236 bp and 584,763 bp includes a number of genes encoding phage tail proteins, as well as transposases and integrases (Fig. 5a). Another 32 kb region (886,091 bp - 918,126 bp) also contains transposase, terminase and integrase encoding genes (Fig. 5d). A 39.4 kb region between 1,564,361 bp and 1,603,857 bp contains genes related to the biosynthesis of tail and head proteins, a protease, portal protein, terminase and integrase (Fig. 5b). A 30.2 kb region between 1,826,924 bp and 1,857,190 bp contains genes encoding a transposase, terminase, portal protein, capsid, head and recombinase. This region also contains an additional gene annotated as *mucBP* (Locus tag: N573_RS03620), which encodes amino acid protein containing 17 MucBP binding domain repeats. However, because of the absence of a cell wall anchor domain required for attachment, it is unlikely that this protein plays a role in adhesion (Fig. 5c). In addition, there are prophage-related genes (not identified by PHAST) adjacent to the bacteriocin encoding region. The prophage-related regions 550,236 bp - 584,763 bp, 1,564,361 bp - 1,603,857 bp and 1,826,924 bp - 1,857,190 have similarities in completely sequenced genomes of the species (Fig. 5a-b), whilst region 749,875 bp - 919,330 bp (containing prophage-related genes between 826,924 bp and 857,190 bp) is unique for strain 3872 (Fig. 5d).

## Conclusion

Completion of the genome sequence of *L. fermentum* 3872 allowed the identification of various features that may contribute to probiotic properties of this bacterium, in addition to the already described CBP-encoding gene carried by the pLF3872 plasmid [9, 10]. Among these is a novel putative bacteriocin-encoding gene not found in any other genomes sequenced to date. Since a gene encoding a putative mucus-binding protein (Locus tag: N573_RS03620) suggests leucine as start codon, it remains to be verified whether the protein is actually expressed. There is a number of other genes (shared with other lactic acid bacteria) potentially required for bacterial attachment to host cells, survival in unfavourable conditions and resistance to toxic compounds. Despite the presence of some conserved features shared by all *L. fermentum* genomes, and a very high similarity between their sequences, the genome of strain 3872 has a large number of unique genes such as *epsH,* and a putative adhesion gene, *inlJ*. A gene that may promote bacterial aggregation has also been found. These genes could be a subject of further investigation. Conservation within the genome of as many as four large prophage-related gene clusters may also contribute to the lifestyle and probiotic properties of this microorganism. In particular, some bacteriocins produced by other bacteria resemble components of bacteriophages, and are encoded by prophage regions of the chromosomes [36]. The bacteriophage-related gene products are being studied as alternatives to antibiotics due to their high potency and specificity, and thus may be of interest for further investigation [35, 37]. As *L. fermentum* 3872 was isolated from the milk of a healthy human female, the presence of multiple vitamin synthesising genes, along with the genes allowing the bacterium to thrive in the gut environment, would make *L. fermentum* an ideal candidate for probiotic studies. The ability of these bacteria to produce various adhesins may allow competitive exclusion of pathogenic microorganisms employing similar mechanisms of adhesion and interacting with the same host cell receptors. The presence of a novel bacteriocin-encoding gene may also contribute to beneficial properties of this strain.

## Additional file

Additional file 1: Table S1. Associated MIGS record. (DOC 72 kb)

## Abbreviations

BASys: Bacterial annotation system
CBP: Collagen-binding protein
GIT: Gastrointestinal tract
HGAP: Hierarchical genome assembly process
MRS agar: Agar developed by de Man, Rogosa and Sharpe
PROKKA: Rapid prokaryotic genome annotation tool

## References

[1] Bubnov RV, Spivak MY, Lazarenko LM, Bomba A, Boyko NV (2015). Probiotics and immunity: provisional role for personalized diets and disease prevention. The EPMA Journal.

[2] Yang G, Liu Z, Yang P (2013). Treatment of allergic rhinitis with probiotics: An alternative approach. North Am J Med Sci.

[3] Azcárate-Peril MA, Sikes M, Bruno-Bárcena JM (2011). The intestinal microbiota, gastrointestinal environment and colorectal cancer: a putative role for probiotics in prevention of colorectal cancer?. Am J Physiol.

[4] Collado MC, Isolauri E, Salminen S, Sanz Y (2009). The impact of probiotic on gut health. Curr Drug Metab.

[5] Jensen H, Dromtorp SM, Axelsson L, Grimmer S (2015). Immunomodulation of Monocytes by Probiotic and Selected Lactic Acid Bacteria. Probiotics and Antimicrobial Proteins.

[6] Tajabadi N, Mardan M, Saari N, Mustafa S, Bahreini R, Mohd Yazid AM (2013). Identification of *Lactobacillus plantarum*, *Lactobacillus pentosus* and *Lactobacillus fermentum* from honey stomach of honeybee. Brazilian J Microbiol.

[7] Anderson RC, Young W, Clerens S, Cookson AL, McCann MJ, Armstrong KM (2013). Human Oral Isolate *Lactobacillus fermentum* AGR1487 Reduces Intestinal Barrier Integrity by Increasing the Turnover of Microtubules in Caco-2 Cells. PLoS One.

[8] Abramov VM, Khlebnikov VS, Pchelintsev SJ, Kosarev IV, Karlyshev AV, Vasilenko RN, Melnikov VG. Strain *Lactobacillus fermentum* having broad spectrum of antagonistic activity and probiotic lactobacterium consortium for manufacturing bacterial preparations” RU 2528862 C1, (20.09.14, Russia). Patent. Application Number: 2013118084/10, Application Date: 19.04.2013, Publication Number: 0002528862, Publication Date: 20.09.2014.

[9] Karlyshev AV, Raju K, Abramov VM. Draft Genome Sequence of *Lactobacillus fermentum* Strain 3872. Genome Announcements. 2013;doi:10.1128/genomeA.01006-13*.* 10.1128/genomeA.01006-13 PMC3869337 24285652

[10] Lehri B, Seddon AM, Karlyshev AV. *Lactobacillus fermentum* 3872 genome sequencing reveals plasmid and chromosomal genes potentially involved in a probiotic activity. FEMS Microbiol Lett. 2015;doi:10.1093/femsle/fnv068 10.1093/femsle/fnv068 25908870

[11] Seemann T. Prokka: rapid prokaryotic genome annotation. Bioinformatics. 2014;doi:10.1093/bioinformatics/btu153 10.1093/bioinformatics/btu153 24642063

[12] Van Domselaar GH, Stothard P, Shrivastava S, Cruz JA, Guo A, Dong X (2005). BASys: a web server for automated bacterial genome annotation. Nucleic Acids Res.

[13] Overbeek R, Olson R, Pusch GD, Olsen GJ, Davis JJ, Disz T (2014). The SEED and the Rapid Annotation of microbial genomes using Subsystems Technology (RAST). Nucleic Acids Res.

[14] Angiuoli SV, Gussman A, Klimke W, Cochrane G, Field D, Garrity GM (2008). Toward an Online Repository of Standard Operating Procedures (SOPs) for (Meta) genomic Annotation. OMICS: J Integr Biol.

[15] Kearse M, Moir R, Wilson A, Stones-Havas S, Cheung M, Sturrock S (2012). Geneious Basic: An integrated and extendable desktop software platform for the organization and analysis of sequence data. Bioinformatics.

[16] Alikhan N, Petty NK, Ben Zakour NL, Beatson SA (2011). BLAST Ring Image Generator (BRIG): simple prokaryote genome comparisons. BMC Genomics.

[17] Grigoriev A (1998). Analyzing genomes with cumulative skew diagrams. Nucleic Acids Res.

[18] Pessione E (2012). Lactic acid bacteria contribution to gut microbiota complexity: lights and shadows. Front Cell Infect Microbiol.

[19] Lee K, Pi K, Kim EB, Rho B, Kang S, Lee HG (2010). Glutathione-mediated response to acid stress in the probiotic bacterium, *Lactobacillus salivarius*. Biotechnol Lett.

[20] Hamon E, Horvatovich P, Marchioni E, Aoude-Werner D, Ennahar S (2014). Investigation of potential markers of acid resistance in *Lactobacillus plantarum* by comparative proteomics. J Appl Microbiol.

[21] El Ghachi M, Derbise A, Bouhss A, Mengin-Lecreulx D (2005). Identification of multiple genes encoding membrane proteins with undecaprenyl pyrophosphate phosphatase (UppP) activity in *Escherichia coli*. J Biol Chem.

[22] Prasad J, McJarrow P, Gopal P (2003). Heat and osmotic stress responses of probiotic *Lactobacillus rhamnosus* HN001 (DR20) in relation to viability after drying. Appl Environ Microbiol.

[23] Izquierdo E, Horvatovich P, Marchioni E, Aoude-Werner D, Sanz Y, Ennahar S (2009). 2-DE and MS analysis of key proteins in the adhesion of *Lactobacillus plantarum*, a first step toward early selection of probiotics based on bacterial biomarkers. Electrophoresis.

[24] Bergonzelli G, Granato D, Pridmore R, Marvin-Guy L, Donnicola D, Corthesy-Theulaz I (2006). GroEL of *Lactobacillus johnsonii* La1 (NCC 533) is cell surface associated: Potential role in interactions with the host and the gastric pathogen Helicobacter pylori. Infect Immun.

[25] Granato D, Bergonzelli G, Pridmore R, Marvin L, Rouvet M, Corthesy-Theulaz I (2004). Cell surface-associated elongation factor Tu mediates the attachment of *Lactobacillus johnsonii* NCC533 (La1) to human intestinal cells and mucins. Infect Immun.

[26] Harris RS. Improved pairwise alignment of genomic DNA. [PhD dissertation]. The Pennsylvania State University; 2007.

[27] Van Heel AJ, de Jong A, Montalban-Lopez M, Kok J, Kuipers OP (2013). BAGEL3: automated identification of genes encoding bacteriocins and (non-)bactericidal posttranslationally modified peptides. Nucleic Acids Res.

[28] Sabet C, Lecuit M, Cabanes D, Cossart P, Bierne H (2005). LPXTG Protein InlJ, a Newly Identified Internalin Involved in Listeria monocytogenes Virulence. Infect Immun.

[29] Juge N (2012). Microbial adhesins to gastrointestinal mucus. Trends Microbiol.

[30] Deivanayagam CC, Rich RL, Carson M, Owens RT, Danthuluri S, Bice T (2000). Novel fold and assembly of the repetitive B region of the *Staphylococcus aureus* collagen-binding surface protein. Structure.

[31] Suessmuth SD, Muscholl-Silberhorn A, Wirth R, Susa M, Marre R, Rozdzinski E (2000). Aggregation Substance Promotes Adherence, Phagocytosis, and Intracellular Survival of *Enterococcus faecalis* within Human Macrophages and Suppresses Respiratory Burst. Infect Immun.

[32] Jones SE, Paynich ML, Kearns DB, Knight KL (2014). Protection from intestinal inflammation by bacterial exopolysaccharides. J Immunol (Baltimore, Md: 1950).

[33] Salzillo M, Vastano V, Capri U, Muscariello L, Sacco M, Marasco R (2015). Identification and characterization of enolase as a collagen-binding protein in *Lactobacillus plantarum*. J Basic Microbiol.

[34] Ozer E, Allen J, Hauser A (2014). Characterization of the core and accessory genomes of *Pseudomonas aeruginosa* using bioinformatic tools Spine and AGEnt. BMC Genomics.

[35] Zhou Y, Liang Y, Lynch KH, Dennis JJ, Wishart DS. PHAST: A Fast Phage Search Tool. Nucleic Acids Research. 2011;doi:10.1093/nar/gkr485 10.1093/nar/gkr485 PMC3125810 21672955

[36] Nakayama K, Takashima K, Ishihara H, Shinomiya T, Kageyama M, Kanaya S (2000). The R-type pyocin of *Pseudomonas aeruginosa* is related to P2 phage, and the F-type is related to lambda phage. Mol Microbiol.

[37] Coates ARM, Hu Y (2007). Novel approaches to developing new antibiotics for bacterial infections. Br J Pharmacol.

[38] Field D, Garrity G, Gray T, Morrison N, Selengut J, Sterk P (2008). The minimum information about a genome sequence (MIGS) specification. Nat Biotechnol.

[39] Woese CR, Kandler O, Wheelis ML. Towards a natural system of organisms: Proposal for the domains Archaea, Bacteria, and Eucarya. Proc Natl Acad Sci. U S A. 1990;87:4576. 10.1073/pnas.87.12.4576 PMC54159 2112744

[40] Gibbons NE, Murray RGE. Proposals Concerning the Higher Taxa of Bacteria. Int J Syst Bacteriol. 1978;28:1–6.

[41] Euzéby: Validation List No. 132 (2010). List of new names and new combinations previously effectively, but not validly, published. Int J Syst Evol Microbiol.

[42] Ludwig W, Schleifer K-H, Whitman WB. Class I. Bacilli class nov. In: Bergey's Manual of Systematic Bacteriology. 2009;3:19–20.

[43] Winslow CEA, Broadhurst J, Buchanan RE, Krumwiede C, Rogers LA, Smith GH (1917). The Families and Genera of the Bacteria: Preliminary Report of the Committee of the Society of American Bacteriologists on Characterization and Classification of Bacterial Types. J Bacteriol.

[44] Skerman VBD, McGowan V, Sneath PHA (1980). Approved Lists of Bacterial Names. Int J Syst Bacteriol.

[45] Beijerinck MW (1901). Sur les ferments lactiques de l'industrie. Archives Néerlandaises des Sciences Exactes et Naturelles.

[46] Ashburner M, Ball CA, Blake JA, Botstein D, Butler H, Cherry JM (2000). Gene ontology: tool for the unification of biology. The Gene Ontology Consortium. Nat Genet.

[47] Edgar RC (2004). MUSCLE: multiple sequence alignment with high accuracy and high throughput. Nucleic Acids Res.

[48] Huelsenbeck JP, Ronquist F (2001). MRBAYES: Bayesian inference of phylogenetic trees. Bioinformatics.

