# Supplementary material for: Potential probiotic-associated traits revealed from completed high quality genome sequence of Lactobacillus fermentum 3872
Source: Stand Genomic Sci. 2017 Feb 1;12:19. doi: 10.1186/s40793-017-0228-4 (PMC5286655; doi:10.1186/s40793-017-0228-4)
Supplement: Additional file 1: Table S1. — Associated MIGS record. (DOC 72 kb) [file 40793_2017_228_MOESM1_ESM.doc]

**Table S1.** Associated MIGS record

| **MIGS-ID** | field name | description |
| --- | --- | --- |
| **MIGS-1** | Submit to INSDC/Trace archives | GenBank, JGI IMG |
| **1.1** | GI | 835164530 |
| **1.2** | Trace Archive |  |
| **MIGS-2** | MIGS CHECK LIST TYPE |  |
| **MIGS-3** | Project Name | [*Lactobacillus fermentum*](http://doi.org/10.1601/nm.5365) 3872 genome sequencing |
| **MIGS-4** | Geographic Location | [Russia](https://www.google.com/maps/place/Russia) |
| **4.1** | Latitude |  |
| **4.2** | Longitude |  |
| **4.3** | Depth |  |
| **4.4** | Altitude |  |
| **MIGS-5** | Time of Sample collection | 2011 |
| **MIGS-6** | Habitat (EnvO) | [*Homo sapiens*](http://www.ncbi.nlm.nih.gov/Taxonomy/Browser/wwwtax.cgi?lvl=0&id=9606); milk |
| **6.1** | temperature | 30-42 °C |
| **6.2** | pH |  |
| **6.3** | salinity |  |
| **6.4** | chlorophyll | none |
| **6.5** | conductivity |  |
| **6.6** | light intensity |  |
| **6.7** | dissolved organic carbon (DOC) |  |
| **6.8** | current |  |
| **6.9** | atmospheric data | Facultative anaerobe |
| **6.10** | density |  |
| **6.11** | alkalinity |  |
| **6.12** | dissolved oxygen |  |
| **6.13** | particulate organic carbon (POC) |  |
| **6.14** | phosphate |  |
| **6.15** | nitrate |  |
| **6.16** | sulfates |  |
| **6.17** | sulfides |  |
| **6.18** | primary production |  |
| **MIGS-7** | Subspecific genetic lineage |  |
| **MIGS-9** | Number of replicons |  |
| **MIGS-10** | Extrachromosomal elements | 1 plasmid |
| **MIGS-11** | Estimated Size |  |
| **MIGS-12** | Reference for biomaterial or Genome report |  |
| **MIGS-13** | Source material identifiers |  |
| **MIGS-14** | Known Pathogenicity | none |
| **MIGS-15** | Biotic Relationship |  |
| **MIGS-16** | Specific Host | [*Homo sapiens*](http://www.ncbi.nlm.nih.gov/Taxonomy/Browser/wwwtax.cgi?lvl=0&id=9606) |
| **MIGS-17** | Host specificity or range (taxid) | 9606 |
| **MIGS-18** | Health status of Host | Healthy |
| **MIGS-19** | Trophic Level |  |
| **MIGS-22** | Relationship to Oxygen | Facultative anaerobe |
| **MIGS-23** | Isolation and Growth conditions | 37°C, anaerobic condition, overnight on MRS agar |
| **MIGS-27** | Nucleic acid preparation | QIAGEN Yst/Bct Kit B DNA extraction kit |
| **MIGS-28** | Library construction | IonTorrent OT2 400bp template preparation kit  SMRTbell template preparation kit |
| **28.1** | Library size | 400bp for IonTorrent PGM, 10Kb for PacBio |
| **28.2** | Number of reads | 423,275 (Ion Torrent run1), 479,672 (Ion Torrent run 2), 387,917 (Ion Torrent run 3), 74,688 (PacBioRsII) |
| **28.3** | vector | None |
| **MIGS-29** | Sequencing method | Next generation sequencing |
| **MIGS-30** | Assembly | Hierarchical genome assembly process (HGAP), MIRA assembler (modified for Ion Torrent reads) |
| **30.1** | Assembly method | De-Novo |
| **30.2** | estimated error rate |  |
| **30.3** | method of calculation |  |
| **MIGS-31** | Finishing strategy | **Optical Map sequence validation, Ion torrent read mapping of four sequencing runs** |
| **31.1** | Status | Complete genome |
| **31.2** | Mean coverage | 19.7 (PacBio), 49.6 (Ion Torrent run1), 60.1 (Ion Torrent run 2),  47.9 (Ion Torrent run 3) |
| **31.3** | contigs | Complete genome |
| **MIGS-32** | Relevant SOPs |  |
| **MIGS-33** | Relevant e-resources |  |
